# Supplementary material for: Stall-Feeding of Sheep on Restricted Grazing: Effects on Performance and Serum Metabolites, Ruminal Fermentation, and Fecal Microbiota
Source: Animals (Basel). 2023 Aug 16;13(16):2644. doi: 10.3390/ani13162644 (PMC10451354; doi:10.3390/ani13162644)
Supplement: Supplementary file 1 [file animals-13-02644-s001.zip › animals-2524932-supplementary.pdf]

animals

MDPI

Supplementary Table S1. Effects of different feeding regimes on composition of fecal bacteria at the phylum of the rectal bacterial communities in Huang-huai lambs

**Phylum**	Treatment			SEM	*P*-value
	**CONT** [1]	**G4H**	**G8H**		
Firmicutes	44.22[b]	52.55[a]	53.71[a]	1.61	0.038
Bacteroidetes	39.71	33.08	32.43	1.42	0.069
Proteobacteria	6.93	3.74	3.87	0.71	0.123
Spirochaetes	2.49	3.29	2.54	0.26	0.43
Kiritimatiellaeota	2.21	1.92	1.87	0.22	0.829
Verrucomicrobia	0.49	0.82	0.83	0.13	0.53
Lentisphaerae	0.66	0.43	1.00	0.11	0.091
Fibrobacteres	0.52	0.65	0.92	0.13	0.488
Actinobacteria	0.67	0.67	0.33	0.16	0.622
Actinobacteria	44.22[b]	52.55[a]	53.71[a]	1.61	0.038

[a,b] Means within a row with different subscripts differ when $P < 0.05$.

[1] CONT, indoor-fed lambs without access to pasture; G4H, indoor-fed lambs with daily access to pasture for four hours; G8H, indoor-fed lambs with daily access to pasture for eight hours.

Supplementary Table S2. Effects of different feeding regimes on composition of fecal bacteria at the genus of the rectal bacterial communities in Huang-huai lambs.

**Item**	Treatment			SEM	*P*-value
	CONT [1]	G4H	G8H		
Ruminococcaceae UCG-005	6.53[b]	10.55[a]	10.73[a]	0.74	0.028
Rikenellaceae RC9 gut group	8.50	7.44	8.49	0.49	0.640
Ruminococcaceae UCG-010	3.86[b]	4.37[b]	7.29[a]	0.56	0.021
Ruminobacter	2.43[a]	0.36[b]	0.33[b]	0.40	0.048
Bacteroides	4.89	5.05	4.94	0.29	0.975
Christensenellaceae R-7 group	2.94[b]	4.96 [a]	4.38 [ab]	0.35	0.052
[Eubacterium] copros-tanoligenes group	4.24	3.56	4.02	0.24	0.528
Prevotella 1	4.50	2.90	2.06	0.54	0.199
Treponema 2	2.28	3.11	2.27	0.26	0.364
Alistipes	1.99	2.63	2.75	0.21	0.319
Succinivibrio	2.15	2.48	2.56	0.55	0.958
Ruminococcaceae UCG-014	2.14	2.81	1.73	0.31	0.411
Ruminococcaceae UCG-013	1.69	2.18	2.17	0.18	0.487
Lachnospiraceae NK4A136 group	1.78	1.81	1.60	0.23	0.934
Prevotellaceae UCG-003	1.49	1.90	1.76	0.20	0.725
Ruminococcaceae UCG-002	1.29	2.04	1.51	0.18	0.252
Phascolarctobacterium	1.56	1.20	1.35	0.12	0.514
Prevotellaceae UCG-001	1.28	1.93	0.84	0.20	0.075
Ruminococcaceae NK4A214 group	1.26	1.28	1.00	0.08	0.338
Ruminococcus 1	0.67	1.04	1.04	0.10	0.218
Other	42.52	36.39	37.19	1.26	0.105

[a,b] Means within a row with different subscripts differ when $P < 0.05$.

[1] CONT, indoor-fed lambs without access to pasture; G4H, indoor-fed lambs with daily access to pasture for four hours; G8H, indoor-fed lambs with daily access to pasture for eight hours.
